# Association of serum uromodulin with diabetic kidney disease: a systematic review and meta-analysis

**DOI:** 10.1186/s12882-024-03854-x

**Published:** 2024-11-24

**Authors:** Shaimaa I. Barr, Eman M. Abd El-Azeem, Sahar S. Bessa, Tarek M. Mohamed

**Affiliations:** 1 Biochemistry Department, Faculty of Science, Ain Shams University, Cairo, Egypt; 2 Internal Medicine Department, Faculty of Medicine, Tanta University, Tanta, Egypt; 3 Biochemistry Division, Chemistry Department, Faculty of Science, Tanta University, Tanta, Egypt

**Keywords:** Uromodulin, Diabetic kidney disease, Meta-analysis

## Abstract

**Background:**

Several studies have investigated the association between the changes of serum uromodulin and diabetic kidney disease (DKD). However, the results are still controversial. Therefore, this meta-analysis was conducted to provide a comprehensive evaluation of the association between serum uromodulin levels and DKD.

**Methods:**

PubMed, Cochrane library, Web of Science, and Scopus were systemically searched following the PRISMA protocol to identify the studies that reported the relationship between serum uromodulin level and DKD. To investigate the association between uromodulin and DKD, a standardized mean difference (SMD) with a 95% confidence interval (CI) was used. When significant heterogeneity was detected (*I*^*2*^ > 50%), sensitivity and subgroup analyses were performed to determine the source of heterogeneity. The quality assessment was determined using the Newcastle-Ottawa scale (NOS), and the publications bias were determined by the funnel plot and Egger’s test.

**Results:**

In total, 6 studies with 1774 patients were included in the final analysis. The random effect model was used. The pooled results showed that the serum uromodulin levels were significantly decreased in patients with DKD (SMD: -0.31; 95% CI: -0.48 to -0.13) (*I*^*2*^ = 45%). Upon applying the sensitivity analysis, it showed (SMD: -0.38; 95% CI: -0.49 to -0.27) (*I*^*2*^ = 3%). Subgroup analysis showed that uromodulin level was significantly decreased in DKD regardless of the region of study, in America (SMD: -0.34; 95% CI: -0.51 to -0.17; *p* < 0.0001), Europe (SMD: -0.54; 95% CI: -1.06 to -0.02; *p* = 0.04), and Asia (SMD: -0.63; 95% CI: -1.15 to -0.11; *p* = 0.02), with stronger predictive value in America and Asia than in Europe. Additionally, uromodulin levels were significantly decreased in both type 1 (SMD: -0.34; 95% CI: -0.51 to -0.17; *p* < 0.0001) and type 2 diabetes (SMD: -0.58; 95% CI: -0.95 to -0.22; *p* = 0.002).

**Conclusion:**

This meta-analysis showed a significant association between low levels of serum uromodulin and DKD. So, it could have a predictive role for DKD. However, its performance varied across subgroup analyses restricted by race and clinical settings. Moreover, further studies are required with a focus on the cut-off value for predicting diagnostic accuracy.

**Supplementary Information:**

The online version contains supplementary material available at 10.1186/s12882-024-03854-x.

## Background

Diabetic kidney disease (DKD) is one of the most serious microvascular complications of long-term untreated hyperglycemia, which represents an important cause of chronic kidney disease (CKD) that frequently leads to end-stage renal disease (ESRD). Pathologically, DKD is characterized by glomerular hypertrophy, sclerosis, interstitial fibrosis, and hyperfiltration [1]. Approximately one-third of type 1 and type 2 diabetic patients develop DKD, and about 50% of those with DKD will progress to ESRD, a condition that is irreversible. Current treatment options are restricted to dialysis or renal replacement [2]. The glomerular filtration rate (GFR) and albumin-to-creatinine ratio (ACR) are well-established biomarkers for the diagnosis of DKD. However, these biomarkers lack sensitivity and have limited predictive value, as it has been reported that advanced pathological changes may occur before the onset of microalbuminuria. Additionally, albuminuria can be influenced by other factors, such as exercise and urinary tract infections, leading to its presence in normal individuals without DKD. Renal biopsy is reported as a gold standard method for DKD diagnosis; however, it is not widely used due to its invasive nature. Consequently, the identification of a novel marker for predicting people at risk for DKD is crucial to preventing progression toward ESRD [3, 4].

Uromodulin (Tamm-Horsfall protein) is a biomarker for kidney tubular function, as it is a kidney-specific protein synthesized mainly by epithelial cells lining the thick ascending limb (TAL) of Henle’s loop and the early distal convoluted tubule (DCT). It is localized to the apical plasma membrane and released into the tubular lumen as a polymeric filament [5]. Uromodulin is the most abundant urinary protein, and it exhibits several functions as it protects against urinary tract infection by binding *type I toxin* of bacteria E. coli [6], preventing kidney stone formation, and regulating water-electrolyte balance through the modulation of the Na+, K+, and 2Cl − cotransporter (NKCC2) and the renal outer medullary K + channel (ROMK2) [7, 8]. Furthermore, studies showed that uromodulin may exhibit a pro- or anti-inflammatory effect [9, 10]. In diabetes, structural alterations in tubular cells, particularly in the TAL, result in tubular damage and a reduction in TAL cell mass [11]. Studies suggested that TAL damage results in the release of uromodulin into the renal interstitial and attenuates the serum concentration of uromodulin [12]. In addition, other studies showed that the excretion of urinary uromodulin decreased in macroalbuminuria compared to normoalbuminuria within the diabetic spectrum [11, 13]. Therefore, decreased serum uromodulin levels may reflect renal damage and reduced renal cell mass [14].

There is a growing interest in investigating the potential role of uromodulin for DKD. Numerous studies have investigated the association between urinary uromodulin and DKD, exploring its potential role as a biomarker for DKD. However, the outcomes across these studies have been inconsistent, and its role in DKD is still controversial. To our knowledge, no review has been conducted to evaluate the association between serum uromodulin and DKD. This meta-analysis is the first to analyze the association between serum uromodulin levels and DKD, aiming to provide a more comprehensive understanding of the predictive role and the relationship between uromodulin and DKD.

## Methods

### Search strategy

This review was conducted according to the Preferred Reporting Items for Systemic Reviews and Meta-analysis (PRISMA) protocol [15]. A systematic search was carried out up to May 30, 2024, across four databases: PubMed, Cochrane Library, Scopus, and Web of Science. English-language articles, without restrictions on publication date, were searched to retrieve the relevant studies examining the association between urinary uromodulin level and DKD. The search terms were indexed in the Medical Subject Headings (MeSH), which are: “Uromodulin,” “Tamm-Horsfall protein,” “Diabetic Nephropathy,” “Diabetic Kidney Disease,” and “Diabetic renal tubular damage”. The literature search formula was as follows: “Uromodulin OR Tamm-Horsfall protein” AND “Diabetic Nephropathy OR Diabetic Kidney Disease OR Diabetic renal tubular damage”. Additionally, a manual search for relevant studies was conducted in the reference lists of the included studies and relevant articles in Google Scholar to ensure comprehensive coverage.

The identification and selection of studies were conducted between May 1, 2024, and May 30, 2024. Two authors independently reviewed the studies for inclusion in the analysis, and any disagreements were resolved through consensus or by consulting a third author.

### Literature screening

Studies were included if they met the following criteria: (1) serum level of uromodulin was determined; (2) the samples were enrolled from diabetic patients; (3) mean ± SD for uromodulin levels between patients with and without DKD were reported; and (4) the level of albumin to creatinine ratio (ACR), glomerular filtration rate (GFR), or albumin excretion rate (AER) in patients with DKD was determined. Studies were excluded according to the following criteria: (1) duplicate studies; (2) review articles; (3) nonoriginal research articles; (4) studies with insufficient data to extract; (5) non-human research; or (6) studies that determined the urine level of uromodulin.

### Data extraction

Data were independently extracted from the included studies by two researchers. If any publication didn’t contain full data to extract, the authors of the study were conducted. Additionally, for studies reporting medians, the data were converted to mean and standard deviation (SD) using the method described in [16]. The following information was included: the first author, the year of publication, country of included studies, type of diabetes, number of participants, age of participants, the detection method for uromodulin, diagnostic criteria for DKD, and the disease condition of DKD.

### Quality assessment

The New Castle-Ottawa scale (NOS) for non-randomized studies was used to assess the risk of bias for the included studies, and this evaluation was performed independently by two researchers. Three aspects were used to assess study quality: (1) selection of the study group (including four domains); (2) comparability of the study group (including one domain); and (3) ascertainment of the outcome of interest (including two domains) [17–19]. Studies with a score of 5 or above were considered high-quality [20].

### Statistical analysis

Meta-analysis was carried out using RevMan software version 5.4 (Cochrane Collaboration, Oxford, UK), while publication bias was assessed with STATA software version 16.0 (Stata Crop LP, College Station, TX). The continuous outcomes for the association between uromodulin and DKD were summarized using standardized mean difference (SMD) with 95% confidence intervals (CI). Heterogeneity among studies was assessed using the *I*^*2*^, with *I*^*2*^ > 50% indicating significant heterogeneity, *I*^*2*^ values of 25–50% indicating modest heterogeneity, and *I*^*2*^ values < 25% indicating low heterogeneity [21]. A random-effects model was applied in cases of significant heterogeneity. Sensitivity analysis was performed to determine heterogeneity, robustness, and consistency of the results. Subgroup analyses were conducted to explore the possible source of heterogeneity based on diabetes type (type 1 or type 2) and region (America, Europe, Asia). Meta-analysis results were presented using a forest plot graph. Publication bias was evaluated by a funnel plot and Egger’s test. The results were considered statistically significant when *p-* value < 0.05.

## Results

### Selection of studies

**Fig. 1:**
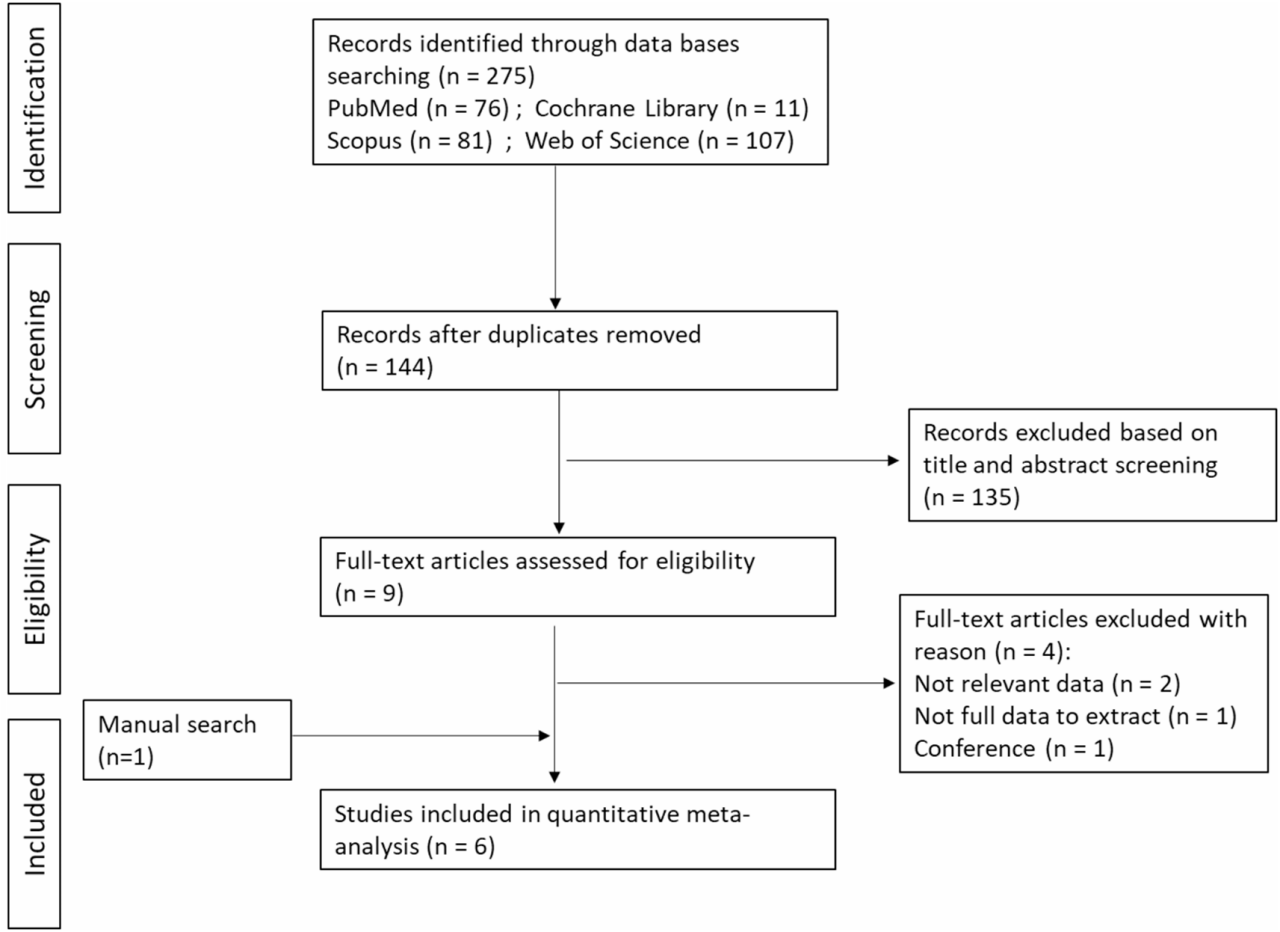
Flowchart of study selection

The systematic search strategy retrieved 275 publications across the 4: PubMed (76), Cochrane Library (11), Web of Science (107), and Scopus (81). After removing duplicates, 144 studies remained. Upon screening the title and abstract, 135 studies were excluded. 9 studies remained for full text and data reviewing. Of these, 4 studies were excluded as they didn’t meet the eligibility criteria. Additionally, 1 study was retrieved through a manual search on Google Scholar. Finally, 6 articles, comprising 1,774 participants, were included in the analysis [22–27]. The detailed screening of studies for the meta-analysis and systematic review are presented in the flow chart (Fig. 1).

### Study characteristics

The main characteristics of the selected studies are summarized in Table 1. The publication year of studies that was eligible for this meta-analysis ranged from 2018 to 2023, with a total of 1,774 participants (862 control, 912 DKD patients). The mean age of participants across the studies varied from 11 to 68 years. 4 studies of them were type 1 diabetes, while 2 studies were type 2 diabetes. The studies were conducted in the following countries: United States (*n* = 2), Canada (*n* = 1), Poland (*n* = 1), Germany (*n* = 1), and Iraq (*n* = 1). Serum uromodulin levels were measured using enzyme-linked immunosorbent assay (ELISA) in 4 studies, and 2 studies were detected by chemiluminescence immunoassay. The enrolled patients were diagnosed with DKD based on eGFR, ACR, or AER values. The quality assessment of all included studies is presented in Table 2, generally; all the included studies were considered to have good quality, as all studies had a quality score > 5, with NOS scores ranged from 6 to 8.

**Table 1:** Characteristics of the included studies

First author	Year	Country	Type of diabetes	No. of participants case (control)	Age case / control	Uromodulin detection method	Diagnostic criteria for DKD	Disease condition of DKD
Bjornstad, P.(a) [22]	2019	United States	type 1	22 / (44)	68 ± 8 / 65 ± 8	Chemiluminescence immunoassay	eGFR < 60 ml/min/1.73m^2^; 24-hour urine albumin excretion > 30 mg/day	eGFR < 60 ml/min/1.73m^2^; 24-hour urine albumin excretion > 30 mg/day
Bjornstad, P.(b) [23]	2019	Canada	type 1	527 / (597)	40 ± 9 / 42 ± 9	Chemiluminescence immunoassay	eGFR < 60 ml/min/1.73m^2^; UACR > 30 mg/g	eGFR < 60 ml/min/1.73m^2^; UACR > 30 mg/g
Ibrahim, A. A [24]	2019	Iraq	type 2	30 / (30)	50.26 ± 1.50 / 48.2 ± 1.69	ELISA	urinary albumin/creatinine ratio (UACR) > 30 mg/g	UACR > 30 mg/g
Schiel, R [25]	2023	Germany	type 1	135 / (69)	11.27 ± 3.35 / 9.68 ± 3.80	ELISA	UACR > 30 mg/g	UACR > 30 mg/g
Wiromrat, P [26]	2019	United States	type 1	179 / (61)	15.2 ± 2.2 / 15.4 ± 2.2	ELISA	UACR > 30 mg/g	UACR > 30 mg/g
Żyłka, A [27]	2018	Poland	type 2	19 / (61)	67 ± 12 / 59 ± 11	ELISA	UACR > 30 mg/g	UACR > 30 mg/g

ELISA: enzyme linked Immunosorbent Assay, DKD: Diabetic kidney disease, eGFR: estimated glomerular filtration rate, UACR: urinary albumin to creatinine ratio

**Table 2:** Quality assessment of New Castle-Ottawa scale (NOS) scale

Reference	Selection	Comparability	Outcome	Evidence quality analysis score of (NOS) out of (8)	Quality assessment
Bjornstad, P.(a) [22]	****	**	**	8	High
Bjornstad, P.(b) [23]	***	*	**	6	High
Ibrahim, A. A [24]	***	**	**	7	High
Schiel, R [25]	****	*	**	7	High
Wiromrat, P [26]	***	**	**	7	High
Żyłka, A [27]	****	*	**	7	High

### Meta-analysis

**Fig. 2:**
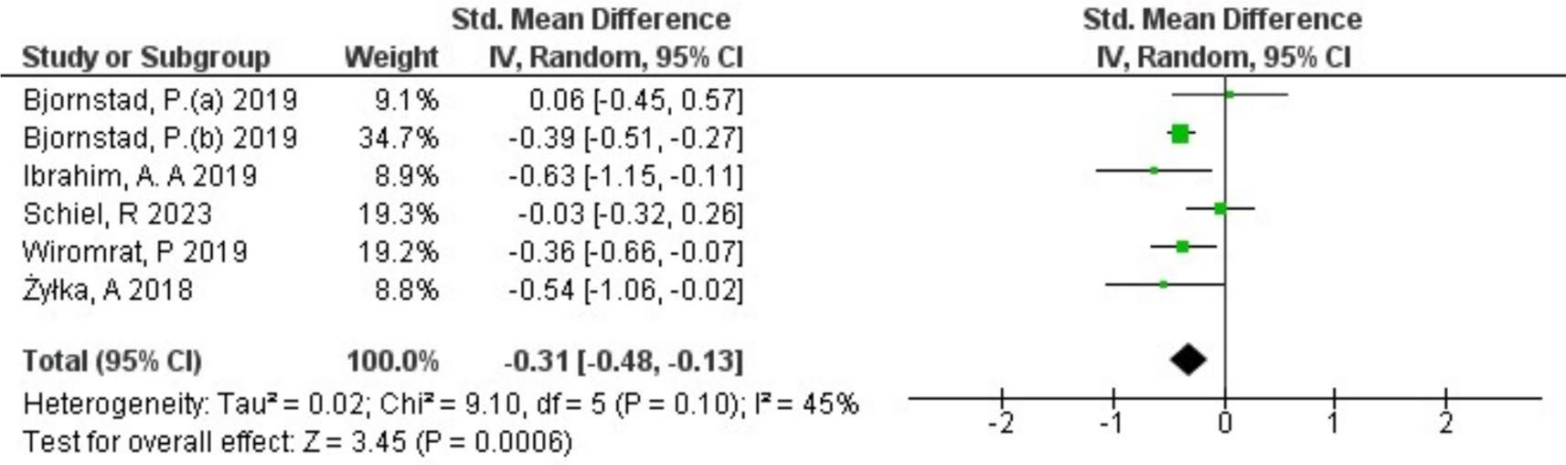
Forest-plot of the meta-analysis of the included studies that investigated the association of uromodulin with DKD compared to control

**Fig. 3:**
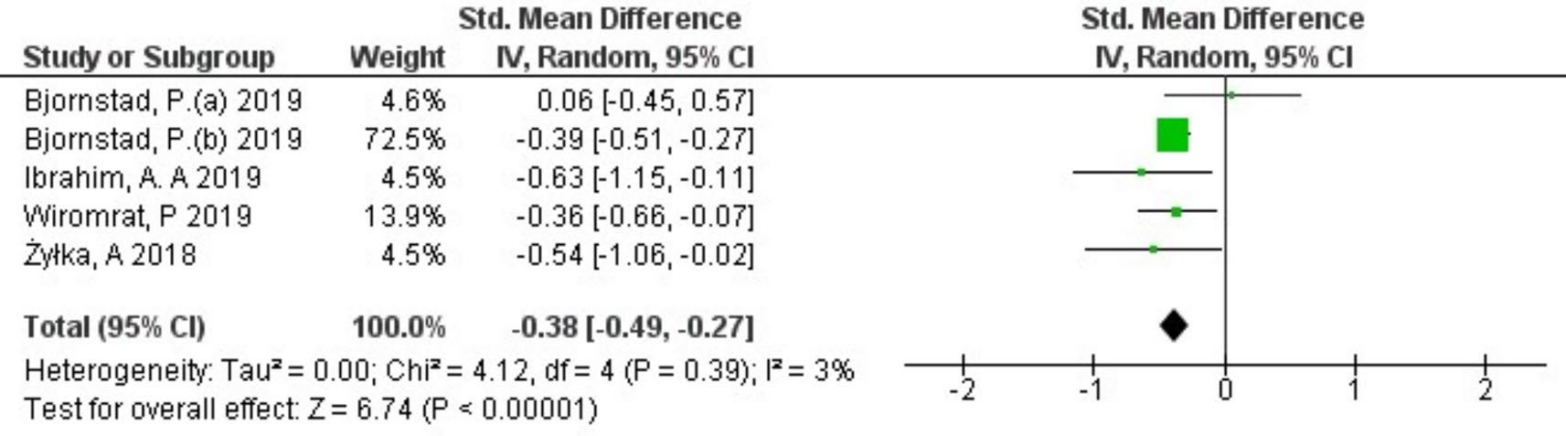
Forest-plot of meta-analysis of the included studies after applying sensitivity analysis

The serum uromodulin levels in patients with and without DKD were reported in all 6 included studies. A randomized-effect model was used as the meta-analysis demonstrated that there was a modest heterogeneity among the included studies (*I*^*2*^ = 45%, *p* = 0.1) (Fig. 2). The pooled results, expressed as SMD with 95% CI, demonstrated a significant decrease in uromodulin levels in DKD patients compared to controls (SMD: -0.31; 95% CI -0.48 to -0.13, *p* < 0.0006) (Fig. 2). To evaluate the heterogeneity and reliability of the results, the sensitivity test was performed by removing each individual study from the pooled results. One study was found to significantly influence the results. After excluding this study, the pooled results (SMD: -0.38; 95% CI -0.49 to -0.27) with low heterogeneity (*I*^*2*^ = 3%, *p* = 0.39) showed a significant decrease in uromodulin level in patients with DKD (*p* < 0.00001) as illustrated in Fig. 3.

**Fig. 4:**
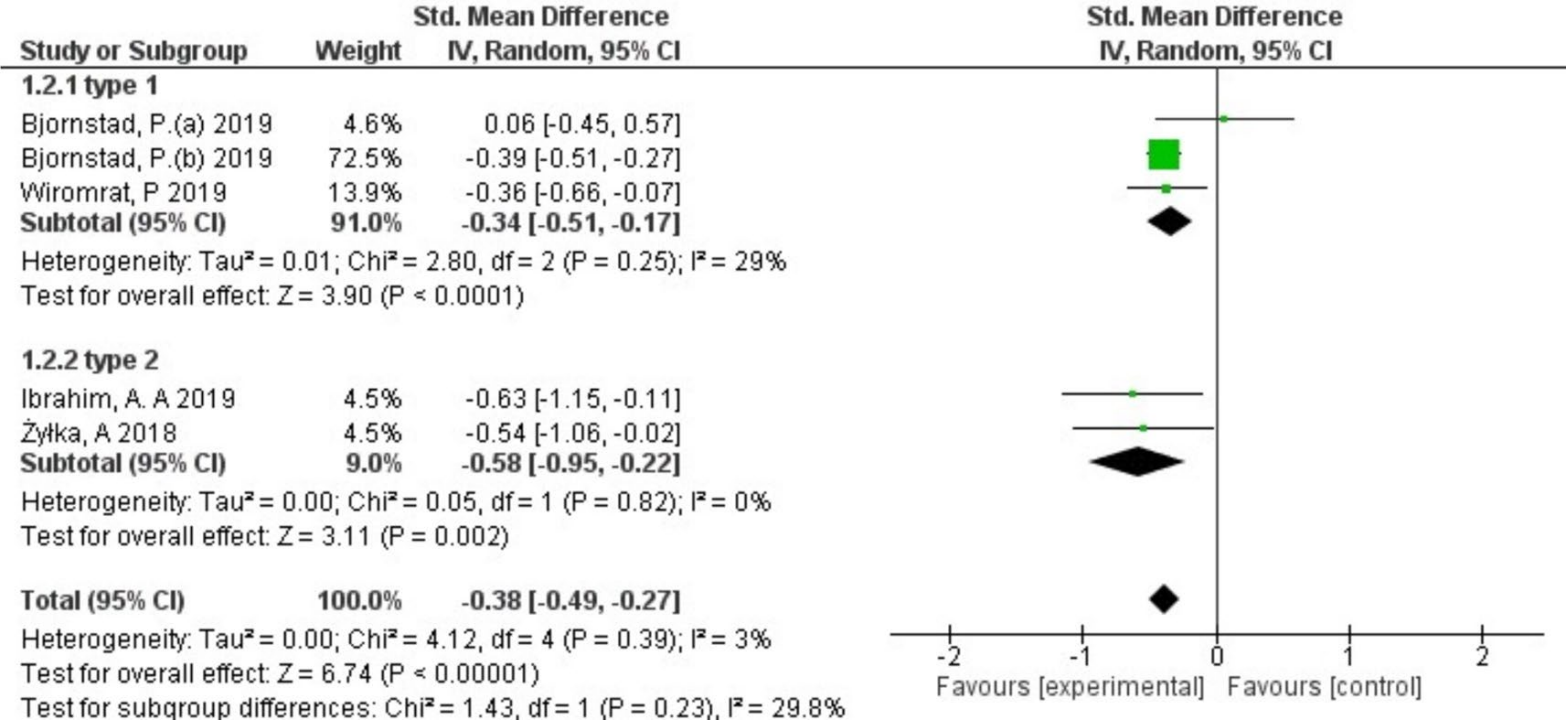
Forest-plot of subgroup meta-analysis according to type of diabetes (type 1, type 2) of the included studies that investigate uromodulin association with DKD compared to control

**Fig. 5:**
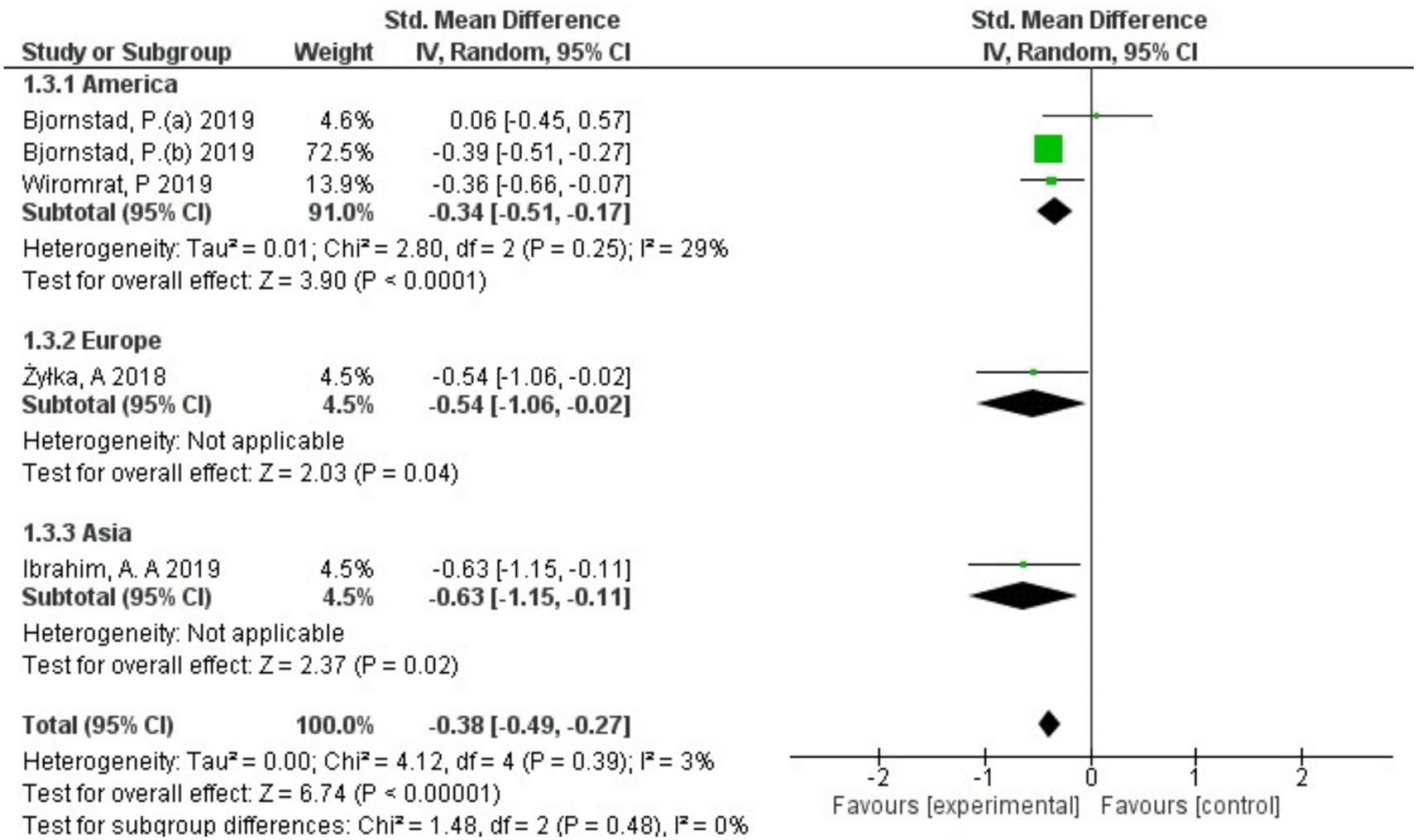
Forest-plot of subgroup meta-analysis according to region (America, Europe, Asia) of the included studies that investigate uromodulin association with DKD compared to control

Subgroup analyses were performed according to type of diabetes and region to determine the source of heterogeneity. Regarding the type of diabetes, the meta-analysis showed that 3 studies were type 1 diabetes with moderate heterogeneity (*I*^*2*^ = 29%) and a pooled SMD of -0.34 (95% CI -0.51 to -0.17), while 2 studies were type 2 diabetes and showed no heterogeneity (*I*^*2*^ = 0%) with a pooled SMD of -0.58 (95% CI -0.95 to -0.22). Moreover, it showed a significant decrease in serum levels of uromodulin with DKD in both type 1 (*p* < 0.0001) and type 2 diabetes (*p* = 0.002) (Fig. 4). In terms of region, America, Europe, and Asia, the meta-analysis showed (SMD: -0.34; 95% CI -0.51 to -0.17) for America; (SMD: -0.54; 95% CI -1.06 to -0.02) for Europe; and for Asia, it showed (SMD: -0.63; 95% CI -1.15 to -0.11) as illustrated in Fig. 5.

**Fig. 6:**
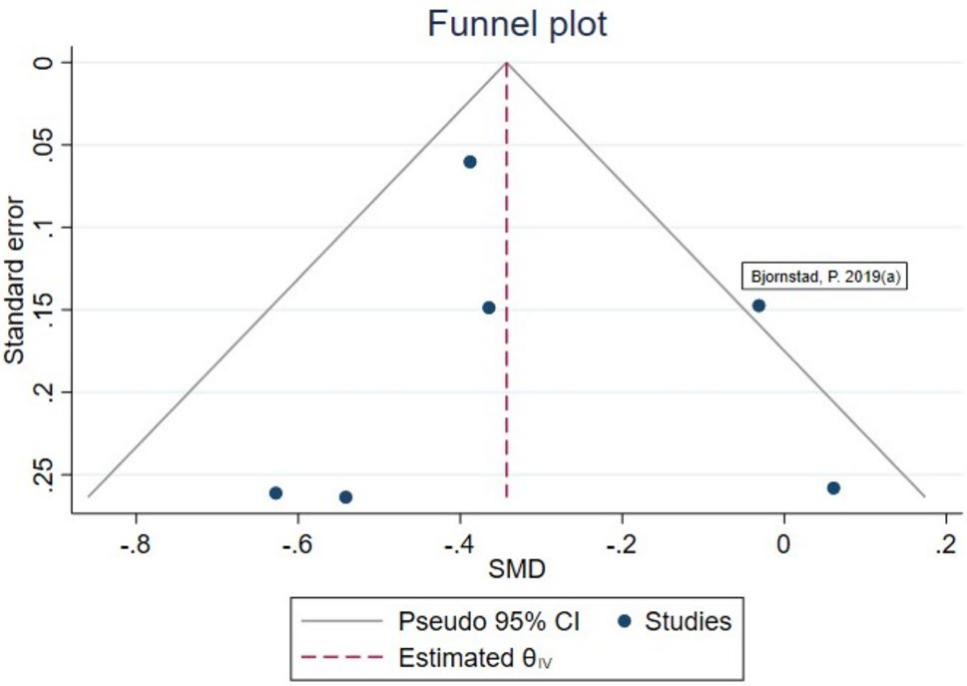
Funnel-plot of publication bias of the included studies

Funnel plot analysis showed an asymmetric shape (Fig. 6), indicating the possibility of publication bias for the association between serum level of uromodulin and DKD. However, the Egger’s test showed that there was no significant evidence of publication bias in this meta-analysis (*p* = 0.95).

## Discussion

Diabetic kidney disease is a complicated disorder that causes significant pathological changes in the structure and function of glomerular and tubular cells, potentially leading to end-stage renal disease (ESRD). The progression of DKD results from the interaction between metabolic and hemodynamic factors, leading to kidney injury, hypertrophy, hyperfiltration, sclerosis, and interstitial fibrosis due to overproduction of profibrotic cytokines and increased reactive oxygen species (ROS) [2]. The gold standard methods for DKD diagnosis are GFR and urinary albumin; however, they lack sensitivity and specificity, as some studies have shown advanced renal cell impairment without changes in urinary albumin levels, while others indicate that microalbuminuria can revert to normoalbuminuria in patients with advanced DKD [28]. Therefore, it is mandatory to identify novel predictors for detecting DKD to reduce the deterioration of kidney function toward ESRD [3].

Uromodulin is a kidney-specific protein mainly synthesized in the thick ascending limb (TAL) of Henel’s loop and may serve as a predictor for assessing tubular function [29]. Uromodulin is produced in response to inflammation associated with interstitial fibrosis, which is the first pathological mechanism in DKD [30]. While the exact role of uromodulin in the inflammatory response remains unclear, some studies suggest it plays a pro-inflammatory role by binding to Toll-like receptor 4 (TLR4) and activating the innate immunity, including monocytes, neutrophils, and dendritic cells, and facilitate neutrophil trans-epithelial migration [9, 30]. Otherwise, some studies indicate an immunosuppressive role by regulating the macrophage inflammatory protein-2 (MIP-2), a potent neutrophil chemoattractant, thereby reducing neutrophil infiltration [31]. Furthermore, it plays a role in the regulation of renal oxidative stress by suppressing the activity of the transient receptor potential cation channel, subfamily M, member 2 (TRPM2) [32]. Additionally, serum uromodulin inhibits vascular calcification by inhibiting cytokine-dependent procalcific signaling [33]. Hyperglycemia induces pathological alterations in tubular cells, leading to TAL damage and a reduction in TAL cell mass [11]. Studies hypothesized that uromodulin reflects the intact tubular mass [26, 34], and TAL damage under hyperglycemic conditions results in uromodulin leakage into the renal interstitial space, consequently attenuating the concentration of serum uromodulin [12]. Thereby, the decreased level of serum uromodulin could reflect the advanced stage and decreased renal cell mass of DKD [14]. However, the role of uromodulin in DKD remains controversial, as the results of individual studies vary. Therefore, this meta-analysis aims to summarize the published results to estimate the predictive role of uromodulin for DKD.

This meta-analysis included data from six studies, revealing a significant association between the reduced concentration of uromodulin and DKD, with concentrations being 0.31 times lower in DKD patients compared to controls (SMD: -0.31; 95% CI: -0.48, -0.13; *p* < 0.0006). Furthermore, after applying the sensitivity analysis to determine the robustness of the pooled results, the overall findings indicated that one study influenced the overall consistency. Upon excluding this study, the heterogeneity was significantly decreased from moderate heterogeneity (*I*^*2*^ = 45%, *p* = 0.1) to low heterogeneity (*I*^*2*^ = 3%, *p* = 0.39), and the pooled results strengthened slightly (SMD: -0.38; 95% CI: -0.49, -0.27; *p* < 0.00001), and demonstrated that the low level of uromodulin is significantly associated with DKD compared to control. This adjustment improved the stability and the robustness of our findings.

In terms of heterogeneity, a subgroup analysis was performed based on the type of diabetes and the region of the studies. Regarding the type of diabetes, modest heterogeneity was observed with type 1 diabetes (*I*^*2*^ = 29%) with a SMD of -0.34 (95% CI: -0.51 to -0.17), while no heterogeneity was present for type 2 diabetes (*I²* = 0%), with an SMD of -0.58 (95% CI: -0.95 to -0.22). This difference may be attributed to the small sample sizes and varying population characteristics. Regarding the region, for America, the meta-analysis showed a SMD of -0.34 (95% CI: -0.51 to -0.17); for Europe, it showed a SMD of -0.54 (95% CI: -1.06 to -0.02); and for Asia, it showed a SMD of -0.63 (95% CI: -1.15 to -0.11) with better predictive role in America and Asia compared to Europe.

The meta-analysis showed that the type of diabetes and region of the population represent sources of heterogeneity, which could be attributed to the limited number of studies, small sample sizes, different races of the population, and differences in the medical care level across different countries. Despite this, the quality assessment and publication bias indicated the consistency and credibility of the results in this meta-analysis.

The small sample size, low number of included studies, variability in the staging and detection methods of DKD, and inclusion of studies from different regions are notable limitations of this study. Furthermore, there is little focus on the cut-off value of uromodulin, making it difficult to predict the diagnostic accuracy of the uromodulin for DKD. Therefore, further studies are required with a focus on the cut-off value of uromodulin to provide the diagnostic potency for the clinical practice of uromodulin for DKD. Nevertheless, this is the first meta-analysis investigating uromodulin in DKD, and the results of this study provide evidence for the predictive role of uromodulin in DKD.

## Conclusion

This meta-analysis suggests that a low level of serum uromodulin is associated with DKD, so it could have a predictive role for DKD. Based on the limitations of this study, further studies with high-quality clinical trials focused on the cut-off value are needed to determine the diagnostic ability and to improve the accuracy and reliability of the findings presented in this study.

## Electronic supplementary material

Below is the link to the electronic supplementary material.

Supplementary Material 1

## Data Availability

All data generated or analyzed during the present study are included in this published article.

## **Declarations**

The authors declared that this manuscript hadn’t been published previously. If it is accepted, it will not be published elsewhere.

### Clinical trial number

NCT06596863.
